# Chlorido{μ-2,6-bis­[(2-amino­eth­yl)imino­meth­yl]-4-chloro­phenolato}-μ-oxido-dicopper(II) trihydrate

**DOI:** 10.1107/S160053681202301X

**Published:** 2012-05-26

**Authors:** Jing-Jing Zhou, Wei Xiao, Jia-Wei Mao, Hong Zhou

**Affiliations:** a Key Laboratory for Green Chemical Processes of the Ministry of Education, Wuhan Institute of Technology, 430073 Wuhan, People’s Republic of China; b Institute of Medicinal Chemistry, Hubei University of Medicine, Shiyan 442000, People’s Republic of China; c College of Chemistry and Molecular Sciences, Wuhan University, Wuhan 430072, People’s Republic of China

## Abstract

In the title dinuclear complex, [Cu_2_(C_14_H_20_ClN_4_O)ClO]·3H_2_O, one Cu^II^ cation assumes a distorted square-planar coordination geometry and the other a distorted square-pyramidal coordination geometry. Both Cu^II^ cations are *N*,*N*′,*O*-chelated by one arm of the 2,6-bis­[(2-amino­eth­yl)imino­meth­yl]-4-chloro­phenolate anion, and one oxide anion bridges the two Cu^II^ cations, forming a dinuclear complex. One of the Cu^II^ cations is further coordinated by an Cl^−^ anion in the apical direction. In the crystal, lattice water mol­ecules are linked with the complex mol­ecule *via* O—H⋯Cl hydrogen bonds while O—H⋯O hydrogen bonding occurs between lattice water mol­ecules , forming three-dimensional network structure.

## Related literature

For the synthesis, see: Gagne *et al.* (1981 ▶). For a related oxygen anion-bridging complex, see: Olmstead *et al.* (2011 ▶). For the biological activity of Schiff bases, see: Raman *et al.* (2007 ▶); Hao *et al.* (2006 ▶). For the biological properties of binuclear complexes, see: Tian *et al.* (2007 ▶); Anbu *et al.* (2009 ▶). Several proteins *in vivo* contain transition metal atoms, especially, Cu^II^, see: Dede *et al.* (2009 ▶); Veysel *et al.* (2003 ▶); Asokan *et al.* (1995 ▶).
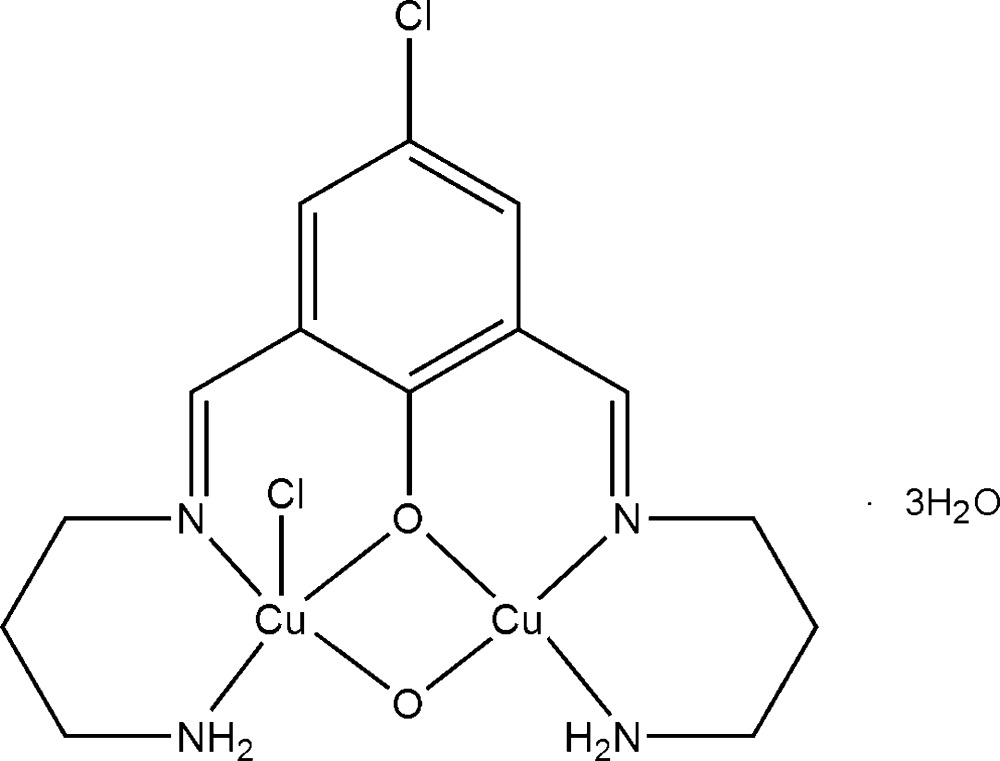


## Experimental

### 

#### Crystal data

[Cu_2_(C_14_H_20_ClN_4_O)ClO]·3H_2_O
*M* *_r_* = 528.37
Monoclinic, 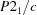

*a* = 11.201 (5) Å
*b* = 12.387 (7) Å
*c* = 16.718 (7) Å
β = 93.18 (4)°
*V* = 2316.0 (19) Å^3^
*Z* = 4
Mo *K*α radiation
μ = 2.10 mm^−1^
*T* = 291 K
0.30 × 0.26 × 0.24 mm

#### Data collection

Bruker SMART APEX CCD diffractometer
Absorption correction: multi-scan (*SADABS*; Bruker, 2000 ▶) *T*_min_ = 0.572, *T*_max_ = 0.633
12029 measured reflections
4087 independent reflections
3286 reflections with *I* > 2σ(*I*)
*R*_int_ = 0.024

#### Refinement

*R*[*F* ^2^ > 2σ(*F* ^2^)] = 0.061
*wR*(*F* ^2^) = 0.175
*S* = 1.02
4087 reflections
244 parameters
H-atom parameters constrained
Δρ_max_ = 0.65 e Å^−3^
Δρ_min_ = −0.94 e Å^−3^

### 

Data collection: *SMART* (Bruker, 2000 ▶); cell refinement: *SAINT* (Bruker, 2000 ▶); data reduction: *SAINT*; program(s) used to solve structure: *SHELXTL* (Sheldrick, 2008) ▶; program(s) used to refine structure: *SHELXTL* ▶; molecular graphics: *SHELXTL* ▶; software used to prepare material for publication: *SHELXTL* ▶.

## Supplementary Material

Crystal structure: contains datablock(s) global, I. DOI: 10.1107/S160053681202301X/xu5529sup1.cif

Structure factors: contains datablock(s) I. DOI: 10.1107/S160053681202301X/xu5529Isup2.hkl

Additional supplementary materials:  crystallographic information; 3D view; checkCIF report

## Figures and Tables

**Table 1:** Hydrogen-bond geometry (Å, °)

*D*—H⋯*A*	*D*—H	H⋯*A*	*D*⋯*A*	*D*—H⋯*A*
O1*W*—H1*WD*⋯Cl1	0.85	1.90	2.583 (12)	136
O2*W*—H2*WB*⋯O3*W*	0.85	2.57	3.114 (13)	123
O3*W*—H3*WD*⋯Cl2	0.85	2.70	3.478 (10)	152

**Fig. 1.**
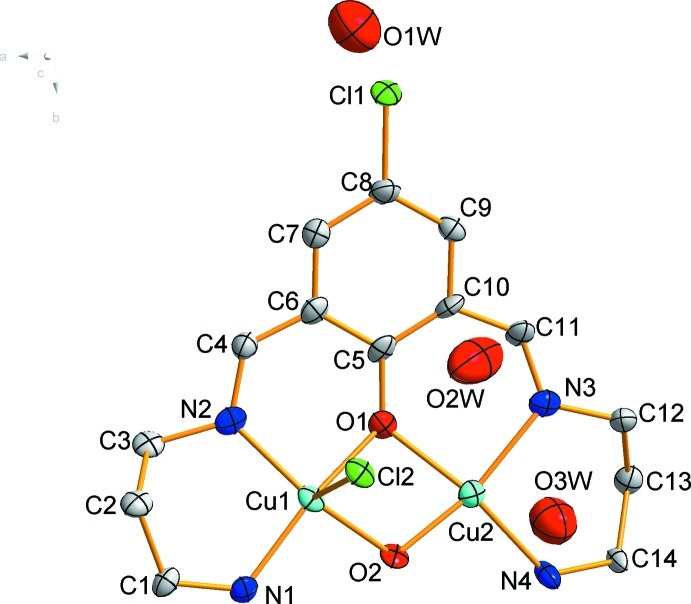
A view of (I), showing the labeling of the non-H atoms and 30% probability ellipsoids. H atoms have been omitted for clarity.

## supplementary crystallographic information

### Comment

Schiff bases have received special attention of biochemists because of their biological activities (Raman *et al.*, 2007; Hao *et al.*, 2006). Researches show that binuclear complexes have good biological properties (Tian *et al.*, 2007; Anbu *et al.*, 2009). Several proteins in vivo contain transition metal centers, especially, the Cu(II) centers (Dede *et al.*, 2009; Veysel *et al.*, 2003). Thus, in this paper, we report on the synthesis and the crystal structure of a new binuclear copper complex. Although a similar complex has been reported in the literature (Asokan *et al.*, 1995), however, the title complex has different substituent in the phenoxid group and counter anion with the reported complex. The crystal structure of the title complex is shown in Fig.1. The molecular unit contains a chloride anion and two copper ions. The coordination environment of the two copper ions are different. Cu2 has four coordinated atoms with quadrilateral configuration, all the atoms locate in an approximately plane with a mean plane derivation of 0.072 Å. While Cu1 has five coordinated atoms with a square pyramid configuration and the chloride anion occupies the apical position with Cu1-Cl2 distance of 2.484 Å. The basal plane is composed of two imino nitrogen atoms, one phenoxide atom and one oxygen anion derived from the solvent of H2O with a mean plane derivation of 0.016 Å. This oxygen anion bridged structure is also found in a dinuclear complex which was obtained by a similar experimental condition (Olmstead *et al.*, 2011).

### Experimental

2,6-Diformyl-4-chlorophenol was prepared according to the literature methods (Gagne *et al.*, 1981). 2,6-Diformyl-4-chlorophenol (0.25 mmol, 0.046 g) in absolute methanol (10 ml) was added to a methanol solution (10 ml) containing 1,3-propanediamine (0.5 mmol, 0.037 g). The solution was stirred vigorously for 2 h at room temperature. Afterwards, a methanol solution (10 ml) of CuCl_2_.2H_2_O (0.5 mmol, 0.085 g) was added dropwise, the mixture was stirred for a further 6 h at ambient temperature. The dark-green block-shaped crystals suitable for X-ray structure analysis were obtained by evaporating the methanol solution of the complex over a period of one month.

### Refinement

H atoms were placed in calculated positions with 0.93–0.97 Å and O—H = 0.85 Å, and included in the refinement in the riding-model approximation, with *U*(H)=1.2–1.5*U*_eq_(C,O).

### Crystal data

**Table:**

[Cu_2_(C_14_H_20_ClN_4_O)ClO]·3H_2_O	*F*(000) = 1080
*M**_r_* = 528.37	*D*_x_ = 1.515 Mg m^−^^3^
Monoclinic, *P*2_1_/*c*	Mo *K*α radiation, λ = 0.71073 Å
Hall symbol: -P 2ybc	Cell parameters from 4001 reflections
*a* = 11.201 (5) Å	θ = 2.4–26.6°
*b* = 12.387 (7) Å	µ = 2.10 mm^−^^1^
*c* = 16.718 (7) Å	*T* = 291 K
β = 93.18 (4)°	Block, green
*V* = 2316.0 (19) Å^3^	0.30 × 0.26 × 0.24 mm
*Z* = 4	

### Data collection

**Table:**

Bruker SMART APEX CCD diffractometer	4087 independent reflections
Radiation source: sealed tube	3286 reflections with *I* > 2σ(*I*)
Graphite monochromator	*R*_int_ = 0.024
phi and ω scans	θ_max_ = 25.0°, θ_min_ = 2.4°
Absorption correction: multi-scan (*SADABS*; Bruker, 2000)	*h* = −13→13
*T*_min_ = 0.572, *T*_max_ = 0.633	*k* = −14→11
12029 measured reflections	*l* = −19→19

### Refinement

**Table:**

Refinement on *F*^2^	Primary atom site location: structure-invariant direct methods
Least-squares matrix: full	Secondary atom site location: difference Fourier map
*R*[*F*^2^ > 2σ(*F*^2^)] = 0.061	Hydrogen site location: inferred from neighbouring sites
*wR*(*F*^2^) = 0.175	H-atom parameters constrained
*S* = 1.02	*w* = 1/[σ^2^(*F*_o_^2^) + (0.080*P*)^2^ + 12.660*P*] where *P* = (*F*_o_^2^ + 2*F*_c_^2^)/3
4087 reflections	(Δ/σ)_max_ = 0.076
244 parameters	Δρ_max_ = 0.65 e Å^−^^3^
0 restraints	Δρ_min_ = −0.94 e Å^−^^3^

### Special details

**Table:**

Geometry. All esds (except the esd in the dihedral angle between two l.s. planes) are estimated using the full covariance matrix. The cell esds are taken into account individually in the estimation of esds in distances, angles and torsion angles; correlations between esds in cell parameters are only used when they are defined by crystal symmetry. An approximate (isotropic) treatment of cell esds is used for estimating esds involving l.s. planes.
Refinement. Refinement of F^2^ against ALL reflections. The weighted R-factor wR and goodness of fit S are based on F^2^, conventional R-factors R are based on F, with F set to zero for negative F^2^. The threshold expression of F^2^ > 2sigma(F^2^) is used only for calculating R-factors(gt) etc. and is not relevant to the choice of reflections for refinement. R-factors based on F^2^ are statistically about twice as large as those based on F, and R- factors based on ALL data will be even larger.

### Fractional atomic coordinates and isotropic or equivalent isotropic displacement parameters (Å^2^)

**Table:**

	*x*	*y*	*z*	*U*_iso_*/*U*_eq_	
Cl1	0.29197 (15)	0.09726 (13)	0.30847 (10)	0.0409 (4)	
Cl2	0.31005 (15)	0.62287 (14)	0.44102 (10)	0.0435 (4)	
Cu1	0.43928 (7)	0.66390 (6)	0.32886 (5)	0.0360 (2)	
Cu2	0.22384 (7)	0.67193 (6)	0.21370 (5)	0.0360 (2)	
C1	0.6375 (6)	0.7673 (6)	0.4411 (4)	0.0388 (15)	
H1A	0.6233	0.8117	0.4874	0.047*	
H1B	0.7089	0.7953	0.4180	0.047*	
C2	0.6665 (7)	0.6538 (6)	0.4715 (4)	0.0451 (17)	
H2A	0.6055	0.6302	0.5066	0.054*	
H2B	0.7427	0.6541	0.5020	0.054*	
C3	0.6721 (6)	0.5750 (5)	0.4003 (4)	0.0376 (15)	
H3A	0.7219	0.6064	0.3607	0.045*	
H3B	0.7101	0.5086	0.4191	0.045*	
C4	0.5264 (6)	0.4435 (6)	0.3522 (4)	0.0395 (15)	
H4	0.5824	0.3950	0.3744	0.047*	
C5	0.3361 (6)	0.4596 (5)	0.2694 (4)	0.0360 (15)	
C6	0.4249 (6)	0.3996 (6)	0.3141 (4)	0.0412 (16)	
C7	0.4106 (6)	0.2905 (6)	0.3267 (4)	0.0358 (14)	
H7	0.4677	0.2524	0.3577	0.043*	
C8	0.3091 (7)	0.2369 (6)	0.2923 (4)	0.0460 (17)	
C9	0.2214 (6)	0.2954 (5)	0.2455 (4)	0.0369 (14)	
H9	0.1554	0.2602	0.2215	0.044*	
C10	0.2354 (6)	0.4051 (5)	0.2363 (4)	0.0396 (15)	
C11	0.1424 (6)	0.4496 (5)	0.1867 (4)	0.0409 (16)	
H11	0.0913	0.3989	0.1621	0.049*	
C12	0.0115 (6)	0.5819 (6)	0.1123 (5)	0.0472 (18)	
H12A	−0.0533	0.6059	0.1440	0.057*	
H12B	−0.0157	0.5176	0.0837	0.057*	
C13	0.0339 (7)	0.6702 (6)	0.0498 (4)	0.0428 (16)	
H13A	−0.0303	0.6710	0.0084	0.051*	
H13B	0.1087	0.6570	0.0249	0.051*	
C14	0.0392 (6)	0.7802 (5)	0.0960 (4)	0.0376 (15)	
H14A	0.0569	0.8361	0.0578	0.045*	
H14B	−0.0403	0.7947	0.1136	0.045*	
N1	0.5352 (5)	0.7852 (5)	0.3811 (3)	0.0398 (13)	
H1C	0.5640	0.8243	0.3411	0.048*	
H1D	0.4827	0.8279	0.4050	0.048*	
N2	0.5521 (5)	0.5481 (5)	0.3605 (3)	0.0417 (13)	
N3	0.1152 (5)	0.5507 (5)	0.1690 (3)	0.0405 (13)	
N4	0.1253 (5)	0.7929 (5)	0.1670 (3)	0.0437 (14)	
H4A	0.0830	0.8191	0.2069	0.052*	
H4B	0.1768	0.8452	0.1545	0.052*	
O1	0.3458 (4)	0.5674 (4)	0.2586 (2)	0.0373 (10)	
O2	0.3320 (4)	0.7525 (3)	0.2702 (3)	0.0360 (10)	
O1W	0.2820 (9)	−0.0067 (9)	0.4419 (7)	0.144 (4)	
H1WD	0.2653	0.0494	0.4142	0.173*	
H1WC	0.2692	0.0055	0.4907	0.216*	
O2W	0.1297 (9)	0.4729 (8)	0.3981 (7)	0.150 (4)	
H2WD	0.1802	0.4946	0.3656	0.180*	
H2WB	0.1094	0.5253	0.4272	0.224*	
O3W	0.0267 (9)	0.7040 (8)	0.3693 (5)	0.120 (3)	
H3WD	0.0988	0.6829	0.3681	0.144*	
H3WA	−0.0136	0.6575	0.3937	0.181*	

### Atomic displacement parameters (Å^2^)

**Table:**

	*U*^11^	*U*^22^	*U*^33^	*U*^12^	*U*^13^	*U*^23^
Cl1	0.0444 (9)	0.0364 (9)	0.0429 (9)	−0.0072 (7)	0.0111 (7)	0.0118 (7)
Cl2	0.0412 (9)	0.0478 (10)	0.0426 (9)	−0.0115 (7)	0.0131 (7)	0.0021 (7)
Cu1	0.0350 (4)	0.0388 (5)	0.0352 (4)	−0.0085 (3)	0.0097 (3)	0.0019 (3)
Cu2	0.0359 (4)	0.0376 (5)	0.0352 (4)	0.0013 (3)	0.0082 (3)	0.0003 (3)
C1	0.038 (3)	0.045 (4)	0.034 (3)	0.009 (3)	0.007 (3)	0.010 (3)
C2	0.047 (4)	0.039 (4)	0.050 (4)	−0.003 (3)	0.014 (3)	0.014 (3)
C3	0.046 (4)	0.036 (3)	0.033 (3)	−0.005 (3)	0.015 (3)	0.016 (3)
C4	0.034 (3)	0.040 (4)	0.044 (4)	0.003 (3)	0.003 (3)	0.005 (3)
C5	0.040 (3)	0.039 (4)	0.030 (3)	0.017 (3)	0.009 (3)	−0.010 (3)
C6	0.038 (4)	0.042 (4)	0.044 (4)	0.012 (3)	0.012 (3)	−0.006 (3)
C7	0.038 (3)	0.043 (4)	0.026 (3)	0.002 (3)	0.006 (3)	0.003 (3)
C8	0.054 (4)	0.032 (4)	0.051 (4)	0.007 (3)	0.000 (3)	−0.008 (3)
C9	0.039 (4)	0.038 (3)	0.035 (3)	−0.007 (3)	0.006 (3)	−0.001 (3)
C10	0.047 (4)	0.028 (3)	0.044 (4)	0.016 (3)	0.000 (3)	0.007 (3)
C11	0.047 (4)	0.035 (4)	0.041 (4)	0.011 (3)	−0.002 (3)	−0.016 (3)
C12	0.038 (4)	0.031 (4)	0.073 (5)	0.002 (3)	−0.003 (3)	−0.008 (3)
C13	0.043 (4)	0.047 (4)	0.038 (4)	0.005 (3)	0.001 (3)	−0.005 (3)
C14	0.038 (3)	0.032 (3)	0.040 (4)	−0.011 (3)	−0.020 (3)	0.017 (3)
N1	0.036 (3)	0.040 (3)	0.044 (3)	0.009 (2)	0.008 (2)	−0.015 (3)
N2	0.047 (3)	0.042 (3)	0.037 (3)	0.007 (3)	0.016 (3)	−0.002 (2)
N3	0.046 (3)	0.039 (3)	0.038 (3)	0.002 (3)	0.014 (2)	−0.007 (2)
N4	0.044 (3)	0.045 (3)	0.040 (3)	−0.020 (3)	−0.014 (3)	0.010 (3)
O1	0.036 (2)	0.043 (3)	0.034 (2)	0.001 (2)	0.0127 (19)	−0.0118 (19)
O2	0.036 (2)	0.032 (2)	0.041 (2)	−0.0120 (19)	0.0114 (19)	0.0163 (19)
O1W	0.139 (9)	0.154 (9)	0.141 (9)	−0.022 (7)	0.014 (7)	−0.009 (7)
O2W	0.133 (8)	0.115 (8)	0.208 (12)	0.016 (6)	0.076 (8)	0.047 (8)
O3W	0.123 (7)	0.119 (7)	0.118 (7)	−0.016 (6)	0.000 (6)	0.015 (6)

### Geometric parameters (Å, º)

**Table:**

Cl1—C8	1.763 (7)	C7—C8	1.411 (10)
Cl2—Cu1	2.484 (2)	C7—H7	0.9300
Cu1—O2	1.865 (4)	C8—C9	1.420 (10)
Cu1—O1	1.941 (4)	C9—C10	1.377 (9)
Cu1—N2	1.965 (6)	C9—H9	0.9300
Cu1—N1	2.017 (6)	C10—C11	1.408 (9)
Cu1—Cu2	3.0040 (18)	C11—N3	1.319 (9)
Cu2—O2	1.797 (4)	C11—H11	0.9300
Cu2—N4	1.994 (6)	C12—N3	1.508 (9)
Cu2—O1	1.997 (5)	C12—C13	1.542 (10)
Cu2—N3	2.048 (6)	C12—H12A	0.9700
C1—N1	1.498 (8)	C12—H12B	0.9700
C1—C2	1.524 (9)	C13—C14	1.565 (9)
C1—H1A	0.9700	C13—H13A	0.9700
C1—H1B	0.9700	C13—H13B	0.9700
C2—C3	1.543 (10)	C14—N4	1.496 (7)
C2—H2A	0.9700	C14—H14A	0.9700
C2—H2B	0.9700	C14—H14B	0.9700
C3—N2	1.503 (9)	N1—H1C	0.9000
C3—H3A	0.9700	N1—H1D	0.9000
C3—H3B	0.9700	N4—H4A	0.9000
C4—N2	1.333 (9)	N4—H4B	0.9000
C4—C6	1.384 (10)	O1W—H1WD	0.8501
C4—H4	0.9300	O1W—H1WC	0.8499
C5—O1	1.353 (8)	O2W—H2WD	0.8496
C5—C10	1.401 (10)	O2W—H2WB	0.8500
C5—C6	1.420 (9)	O3W—H3WD	0.8500
C6—C7	1.378 (10)	O3W—H3WA	0.8501
			
O2—Cu1—O1	74.6 (2)	C7—C8—C9	120.1 (6)
O2—Cu1—N2	163.2 (2)	C7—C8—Cl1	119.4 (5)
O1—Cu1—N2	91.8 (2)	C9—C8—Cl1	120.5 (5)
O2—Cu1—N1	95.8 (2)	C10—C9—C8	119.0 (6)
O1—Cu1—N1	167.6 (2)	C10—C9—H9	120.5
N2—Cu1—N1	96.2 (2)	C8—C9—H9	120.5
O2—Cu1—Cl2	97.57 (13)	C9—C10—C5	121.7 (6)
O1—Cu1—Cl2	90.79 (13)	C9—C10—C11	111.5 (6)
N2—Cu1—Cl2	92.30 (16)	C5—C10—C11	126.7 (6)
N1—Cu1—Cl2	98.30 (17)	N3—C11—C10	131.2 (7)
O2—Cu1—Cu2	34.16 (13)	N3—C11—H11	114.4
O1—Cu1—Cu2	41.00 (14)	C10—C11—H11	114.4
N2—Cu1—Cu2	132.74 (18)	N3—C12—C13	117.3 (6)
N1—Cu1—Cu2	129.95 (17)	N3—C12—H12A	108.0
Cl2—Cu1—Cu2	90.55 (6)	C13—C12—H12A	108.0
O2—Cu2—N4	97.5 (2)	N3—C12—H12B	108.0
O2—Cu2—O1	74.66 (19)	C13—C12—H12B	108.0
N4—Cu2—O1	170.4 (2)	H12A—C12—H12B	107.2
O2—Cu2—N3	165.6 (2)	C12—C13—C14	106.7 (5)
N4—Cu2—N3	95.9 (2)	C12—C13—H13A	110.4
O1—Cu2—N3	92.4 (2)	C14—C13—H13A	110.4
O2—Cu2—Cu1	35.64 (14)	C12—C13—H13B	110.4
N4—Cu2—Cu1	133.09 (16)	C14—C13—H13B	110.4
O1—Cu2—Cu1	39.61 (13)	H13A—C13—H13B	108.6
N3—Cu2—Cu1	130.51 (17)	N4—C14—C13	119.2 (5)
N1—C1—C2	120.0 (6)	N4—C14—H14A	107.5
N1—C1—H1A	107.3	C13—C14—H14A	107.5
C2—C1—H1A	107.3	N4—C14—H14B	107.5
N1—C1—H1B	107.3	C13—C14—H14B	107.5
C2—C1—H1B	107.3	H14A—C14—H14B	107.0
H1A—C1—H1B	106.9	C1—N1—Cu1	123.3 (4)
C1—C2—C3	110.1 (6)	C1—N1—H1C	106.5
C1—C2—H2A	109.6	Cu1—N1—H1C	106.5
C3—C2—H2A	109.6	C1—N1—H1D	106.5
C1—C2—H2B	109.6	Cu1—N1—H1D	106.5
C3—C2—H2B	109.6	H1C—N1—H1D	106.5
H2A—C2—H2B	108.2	C4—N2—C3	116.4 (6)
N2—C3—C2	114.1 (5)	C4—N2—Cu1	123.3 (5)
N2—C3—H3A	108.7	C3—N2—Cu1	120.2 (4)
C2—C3—H3A	108.7	C11—N3—C12	123.0 (6)
N2—C3—H3B	108.7	C11—N3—Cu2	119.3 (5)
C2—C3—H3B	108.7	C12—N3—Cu2	117.5 (4)
H3A—C3—H3B	107.6	C14—N4—Cu2	123.4 (4)
N2—C4—C6	126.7 (6)	C14—N4—H4A	106.5
N2—C4—H4	116.7	Cu2—N4—H4A	106.5
C6—C4—H4	116.7	C14—N4—H4B	106.5
O1—C5—C10	119.5 (6)	Cu2—N4—H4B	106.5
O1—C5—C6	121.8 (6)	H4A—N4—H4B	106.5
C10—C5—C6	118.7 (6)	C5—O1—Cu1	124.8 (4)
C7—C6—C4	114.4 (6)	C5—O1—Cu2	129.2 (4)
C7—C6—C5	120.6 (7)	Cu1—O1—Cu2	99.4 (2)
C4—C6—C5	124.8 (7)	Cu2—O2—Cu1	110.2 (2)
C6—C7—C8	119.8 (6)	H1WD—O1W—H1WC	109.5
C6—C7—H7	120.1	H2WD—O2W—H2WB	109.5
C8—C7—H7	120.1	H3WD—O3W—H3WA	109.5

### Hydrogen-bond geometry (Å, º)

**Table:**

*D*—H···*A*	*D*—H	H···*A*	*D*···*A*	*D*—H···*A*
O1*W*—H1*WD*···Cl1	0.85	1.90	2.583 (12)	136
O2*W*—H2*WB*···O3*W*	0.85	2.57	3.114 (13)	123
O3*W*—H3*WD*···Cl2	0.85	2.70	3.478 (10)	152
